# Artifleck: The Study of Artifactual Responses to Light Flecks with Inappropriate Leaves

**DOI:** 10.3390/plants9070905

**Published:** 2020-07-17

**Authors:** Thomas E. Marler

**Affiliations:** Western Pacific Tropical Research Center, College of Natural and Applied Sciences, University of Guam, Mangilao, GU 96923, USA; marler.uog@gmail.com

**Keywords:** cycad, corn, *Cycas micronesica*, *Glycine max*, light fleck, maize, soybean, *Zea mays*

## Abstract

Methods in sunfleck research commonly employ the use of experimental leaves which were constructed in homogeneous light. These experimental organs may behave unnaturally when they are challenged with fluctuating light. Photosynthetic responses to heterogeneous light and leaf macronutrient relations were determined for *Cycas micronesica*, *Glycine max*, and *Zea mays* leaves that were grown in homogeneous shade, heterogeneous shade, or full sun. The speed of priming where one light fleck increased the photosynthesis during a subsequent light fleck was greatest for the leaves grown in heterogeneous shade. The rate of induction and the ultimate steady-state photosynthesis were greater for the leaves that were grown in heterogeneous shade versus the leaves grown in homogeneous shade. The leaf mass per area, macronutrient concentration, and macronutrient stoichiometry were also influenced by the shade treatments. The amplitude and direction in which the three developmental light treatments influenced the response variables were not universal among the three model species. The results indicate that the historical practice of using experimental leaves which were constructed under homogeneous light to study leaf responses to fluctuating light may produce artifacts that generate dubious interpretations.

## 1. Introduction

The conversion of light energy into biomass through plant photosynthesis has been aggressively studied for decades. The majority of the published research addressing the limitations of photosynthesis has focused on steady-state gas exchange behaviors under the conditions of constant photosynthetic photon flux density (PPFD). However, the incident light that most leaves experience is not supplied as a constant abiotic resource. Alternatively, a continually changing PPFD occurs due to factors such as ephemeral cloud cover, atmospheric conditions that define the extent of diffuse light, the movement of leaves, the size and stratification of canopy foliage gaps, and the changes in angle of the solar beam throughout a photoperiod [1,2,3]. This chronically changing PPFD places the photosynthetic machinery under conditions that do not conform to the experimental methods in studies which employ constant PPFD leading to steady-state photosynthesis.

The stochastic nature of duration, timing, amplitude, PPFD, and the frequency of incident light changes places the photosynthetic machinery of many plants in a chronic state of induction and relaxation [4,5]. The studies of leaf behaviors in fluctuating light have shown that photosynthesis in steady-state conditions is not correlated with the induction traits that result from fluctuating light conditions [6]. Therefore, the improvement of crop performance should include the study of steady-state photosynthetic capacity, but also the photosynthetic traits that define leaf performance in fluctuating light [7,8].

The importance of sunflecks for understory plants has been of interest to ecologists for about one century [9]. However, the direct study of rapid gas exchange responses to sunflecks to answer ecological questions was not possible until the development of fast responding infrared gas analyzers, so this literature has been accumulating since the 1990s [10]. The considerable literature that has amassed contains two common response variables: (1) leaf gas exchange responses to sequentially alternating shade flecks and light flecks, and (2) photosynthetic induction traits following an abrupt transition from limiting PPFD to non-limiting PPFD. For the published controlled laboratory or nursery studies, a common experimental approach has been to grow the plants in homogeneous PPFD conditions prior to the dates of data collection. Experimental methods must adhere to standards of relevance in order for the outcomes to be valid. Considering the stomatal, light-harvesting, and biochemical choreography that controls a leaf’s induction and relaxation responses to fluctuating light, an unnatural leaf that never experienced fluctuating light during its emergence and maturation will not likely exhibit the responses to fluctuating light that align with those of leaves that emerged and matured under ecologically relevant conditions of fluctuating light. Therefore, an artifactual response to experimentally imposed light flecks by a leaf that never experienced an abrupt transition of PPFD prior to the experimenter’s imposition of the experimental light fleck onto the lamina should be expected.

I used the model plant *Glycine max* (L.) Merr. (hereinafter soybean) to represent eudicotyledons, *Zea mays* L. (hereinafter maize) to represent monocotyledons, and *Cycas micronesica* K.D. Hill (hereinafter cycad) to represent gymnosperms, to investigate how incident light throughout a leaf’s construction and maturation influenced leaf physiology during the light-fleck experimental methods. Maize and soybean were selected because of their prominent position as leading crops of the world [11,12]. *Cycas micronesica* was selected because the gas exchange physiology of this species has been studied more so than other cycads [13,14]. Moreover, the literature on the leaf physiology of maize [15,16,17,18], soybean [6,19,20,21,22,23,24,25], and cycad [26] under the conditions of fluctuating light provide a foundation for this expanded knowledge.

My objectives were to determine the influence of the developmental light conditions of experimental leaves on their responses to alternating shade-fleck and light-fleck transitions, photosynthetic induction following a transition from limiting to non-limiting PPFD, leaf mass per area (LMA), and leaf macronutrient concentrations. I predicted that shade-grown leaves that developed under fluctuating light would exhibit induction and priming behaviors that differed from the shade-grown leaves that developed under homogeneous shade-screen conditions. For the purposes of this study, the shade flecks were ephemeral periods of limiting light conditions, light flecks were ephemeral periods of non-limiting light conditions, induction was the process whereby the leaf’s net CO_2_ assimilation gradually increased to a new steady-state following an abrupt increase in PPFD, and priming was considered the ability of one light fleck to increase a leaf’s induction status during a subsequent light fleck.

## 2. Results

### 2.1. Light Flecks

**Figure 1:**
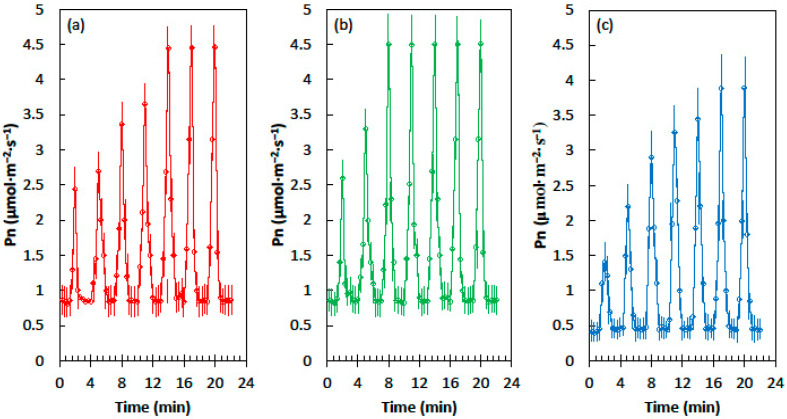
Net CO_2_ assimilation (Pn) of the *Cycas micronesica* leaves in response to the alternating shade flecks with a photosynthetic photon flux density (PPFD) of 55 µmol·m^−2^·s^−1^ and the light flecks with a PPFD of 800 µmol·m^−2^·s^−1^. The experimental leaves were grown in (**a**) homogeneous 62% sunlight exclusion under a shade screen, (**b**) heterogeneous 62% sunlight exclusion under a slat-house, or (**c**) full sun. Each marker is a mean of four replications.

Cycad leaves, developed in homogeneous shade conditions of 62% light exclusion, exhibited minimum and maximum net CO_2_ assimilation (Pn) in fluctuating light that was similar to the leaves that developed in heterogeneous shade of 62% light exclusion (Figure 1). However, priming was more rapid in the leaves that developed in heterogeneous shade. The shade leaves from the homogeneous light treatment required five sequential light flecks to reach the maximum Pn, but the leaves from the heterogeneous light treatment reached maximum Pn after only three light flecks. The Pn of cycad leaves that developed in full sun conditions was lower than the Pn of the shade treatments in the shade flecks and the light flecks (Figure 1c). Moreover, the speed of priming was slow, with six light flecks required before the maximum Pn was reached.

**Figure 2:**
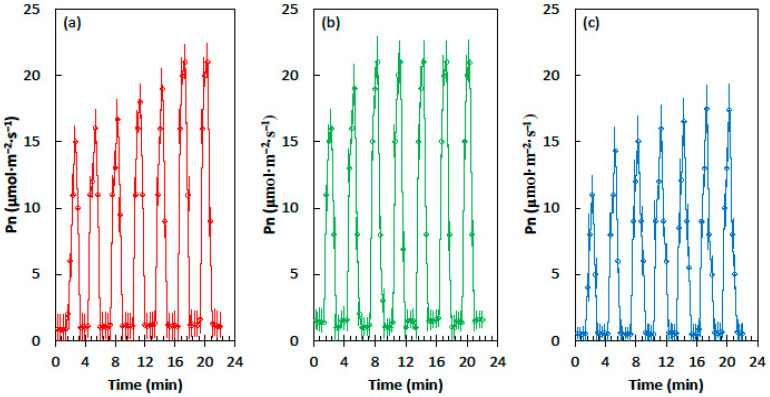
Net CO_2_ assimilation (Pn) of the *Zea mays* leaves in response to the alternating shade flecks with a photosynthetic photon flux density (PPFD) of 55 µmol·m^−2^·s^−1^ and the light flecks with a PPFD of 800 µmol·m^−2^·s^−1^. The experimental leaves were grown in (**a**) homogeneous 62% sunlight exclusion under a shade screen, (**b**) heterogeneous 62% sunlight exclusion under a slat-house, or (**c**) full sun. Each marker is a mean of four replications.

The relationships among the three developmental light treatments for maize leaves was similar to those of cycad leaves, but the Pn values were much greater (Figure 2). Leaves from the homogeneous shade treatment required six sequential light flecks to reach maximum Pn, but the leaves from the heterogeneous shade treatment reached maximum Pn after only three light flecks. The Pn of the corn leaves that developed in full sun conditions was lower than the Pn of the shade treatments in the shade flecks and the light flecks (Figure 2c). The influence of priming was similar to that of the homogeneous shade treatment, as six light flecks were required to reach the maximum Pn.

**Figure 3:**
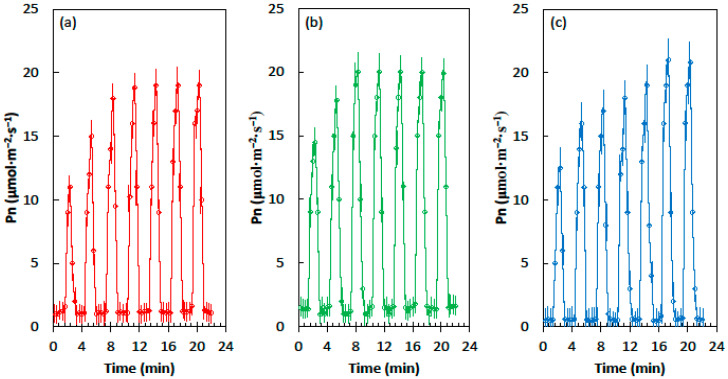
Net CO_2_ assimilation (Pn) of the *Glycine max* leaves in response to the alternating shade flecks with a photosynthetic photon flux density (PPFD) of 55 µmol·m^−2^·s^−1^ and the light flecks with a PPFD of 800 µmol·m^−2^·s^−1^. The experimental leaves were grown in (**a**) homogeneous 62% sunlight exclusion under a shade screen, (**b**) heterogeneous 62% sunlight exclusion under a slat-house, or (**c**) full sun. Each marker is a mean of four replications.

The influence of the developmental light conditions on the Pn of soybean leaves differed from that of cycad and corn leaves. The leaves from the homogeneous shade treatment reached a maximum Pn of about 19 µmol·m^−2^·s^−1^ after four light flecks, the leaves from heterogeneous shade treatment reached a maximum Pn of about 20 µmol·m^−2^·s^−1^ after three light flecks, and the leaves from the full sun treatment reached a maximum Pn of about 21 µmol·m^−2^·s^−1^ after six light flecks (Figure 3).

### 2.2. Photosynthetic Induction

**Figure 4:**
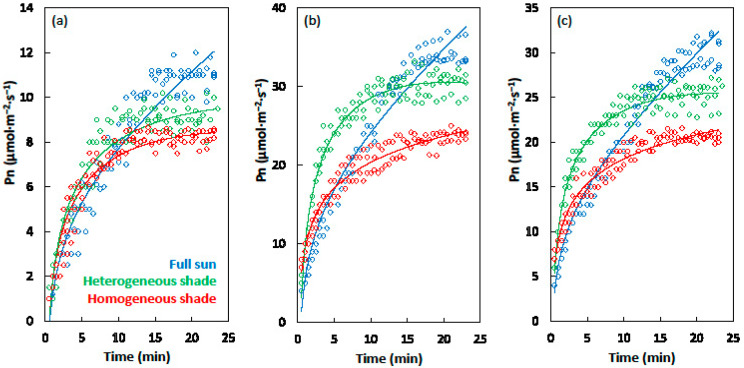
The increase in the net CO_2_ assimilation (Pn) immediately following the supply of the photosynthetic photon flux density of 800 µmol·m^−2^·s^−1^ to the leaves of (**a**) *Cycas micronesica*, (**b**) *Zea mays*, and (**c**) *Glycine max*. For each species, the leaves were grown in homogeneous 62% sunlight exclusion under a shade screen, heterogeneous 62% sunlight exclusion under a slat-house, or full sunlight. The models for each species were: (**a**) **Full sun:** Pn = 0.97 + (0.20 × time) + (2.05 × log(time)); *p* ≤ 0.001; *R*^2^ = 0.94, (**a**) **Full sun**: Pn = 0.97 + (0.20 × time) + (2.05 × log(time)); *p* < 0.001; *R*^2^ = 0.94, (**a**) **Homogeneous shade:** Pn = 1.87 + (−0.10 × time) + (2.85 × log(time)); *p* ≤ 0.001; *R*^2^ = 0.93, (**b**) **Full sun:** Pn = 4.78 + (0.69 × time) + (5.42 × log(time)); *p* ≤ 0.001; *R*^2^ = 0.96, (**b**) **Heterogeneous shade:** Pn = 10.36 + (−0.50 × time) + (10.05 × log(time)); *p* ≤ 0.001; *R*^2^ = 0.96, (**b**) **Homogeneous shade**: Pn = 9.47 + (0.01 × time) + (4.69 × log(time)); *p* < 0.001; *R*^2^ = 0.95, (**c**) **Full sun**: Pn = 5.51 + (0.66 × time) + (3.77 × log(time)); *p* < 0.001; *R*^2^ = 0.97, (**c**) **Heterogeneous shade**: Pn = 10.37 + (−0.30 × time) + (7.00 × log(time)); *p* < 0.001; *R*^2^ = 0.95, (**c**) **Homogeneous shade:** Pn = 8.91 + (−0.01 × time) + (4.05 × log(time)); *p* ≤ 0.001; *R*^2^ = 0.95.

The influence of the developmental light conditions on the induction of photosynthesis followed similar patterns for the three model species (Figure 4). During the first 5 min of induction, Pn exhibited increased values in the order full sun < homogeneous shade < heterogeneous shade. After 10–15 min of induction, the Pn exhibited increased values in the order homogeneous shade < heterogeneous shade < full sun.

The three nonlinear model coefficients that described the induction of photosynthesis were not different for the cycad leaves that developed in the two shade treatments (Table 1). In contrast, all three model coefficients were not the same for the full sun leaves when compared with the corresponding coefficients for the shade-grown leaves. The influence of developmental light conditions on the induction of photosynthesis was similar for the maize and soybean leaves (Table 1). All three model coefficients for the leaves grown in heterogeneous shade exhibited greater values than the leaves grown in homogeneous shade. The coefficient a of full sun leaves was higher and the coefficient b of full sun leaves was lower than that for either shade treatment. In contrast, the coefficient c of full sun leaves did not differ from that of homogeneous shade-grown leaves for both species.

The maximum net CO_2_ assimilation (A_max_) was influenced by developmental light similarly for the three species, with A_max_ increasing in the order homogeneous shade < heterogeneous shade < full sun (Table 1). The time required to reach 50% of full induction (IT 50%) was also influenced by developmental light similarly for the three species, with the full sun leaves requiring more time than the shade leaves, and the two shade treatments exhibiting similar times. The time required to reach 90% of full induction (IT 90%) was influenced by the developmental light similarly for the three species, with IT 90% increasing in the order heterogeneous shade < homogeneous shade < full sun.

### 2.3. Leaf Nutrient Relations

Developmental light conditions did not influence the leaf carbon, potassium, or magnesium concentrations of cycad leaves (Table 2). The leaves that developed in full sun exhibited less nitrogen, calcium, and sulfur than at least one of the shade-treatment leaves. In contrast, the leaves that developed in full sun exhibited greater phosphorus than at least one of the shade-treatment leaves. The leaves in the two shade treatments differed in the leaf concentrations for nitrogen, phosphorus, and sulfur. The leaves in the two shade treatments exhibited leaf concentrations of carbon, potassium, and calcium that were not different. The carbon/nitrogen quotient increased in value in the order homogeneous shade < heterogeneous shade < full sun. The quotients carbon/phosphorus and carbon/potassium did not differ among the three developmental light treatments.

Carbon concentration was not influenced for the maize leaves, but the remainder of the element response variables were influenced by developmental light conditions (Table 3). The leaves that developed in full sun exhibited less nitrogen, phosphorus, potassium, calcium, and sulfur than at least one of the shade-treatment leaves. In contrast, the leaves that developed in full sun exhibited greater magnesium than at least one of the shade-treatment leaves. The leaves in the homogeneous shade treatment differed from those in the heterogeneous shade treatment for every element response variable except carbon. All three stoichiometry variables increased in value in the order homogeneous shade < heterogeneous shade < full sun.

Developmental light conditions did not influence the leaf carbon, calcium, or magnesium concentrations of the soybean leaves (Table 4). The leaves that developed in full sun exhibited less nitrogen and potassium than at least one of the shade-treatment leaves. In contrast, the leaves that developed in full sun exhibited greater phosphorus and sulfur than at least one of the shade-treatment leaves. The leaves in the two shade treatments differed in the leaf concentrations for nitrogen, phosphorus, potassium, and sulfur. The carbon/nitrogen quotient was the least in homogeneous shade, and did not differ between the heterogeneous shade and full sun treatments. The quotients carbon/phosphorus and carbon/potassium did not differ among the three developmental light treatments.

### 2.4. Leaf Mass per Area

**Figure 5:**
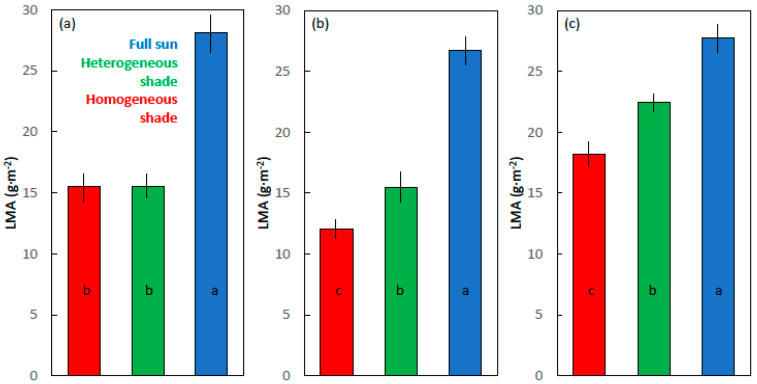
Leaf mass per area (LMA) of the (**a**) *Cycas micronesica*, (**b**) *Zea mays*, and the (**c**) *Glycine max* leaves. For each species, the leaves were grown in homogeneous 62% sunlight exclusion under a shade screen, heterogeneous 62% sunlight exclusion under a slat-house, or full sunlight. Bars with the same letter within each species are not different according to Tukey’s honest significant difference test. Mean ± SE, n = 4.

The LMA ranged from 12 to 18 g·m^−2^ for the homogeneous shade leaves, 15–23 g·m^−2^ for the heterogeneous shade leaves, and 26–28 g·m^−2^ for the full sun leaves (Figure 5). The developmental light treatments influenced the LMA of the cycad leaves (*F*_2,9_ = 28.928; *p* < 0.001), and the full sun leaves exhibited a greater LMA than the two shade treatments. The developmental light treatments influenced the LMA of maize (*F*_2,9_ = 361.731; *p* < 0.001) and soybean (*F*_2,9_ = 99.192; *p* < 0.001) leaves, with the LMA increasing in value in the order homogeneous shade < heterogeneous shade < full sun.

## 3. Discussion

The cumulative literature on the photosynthetic induction and relaxation in fluctuating PPFD has collectively shown that the steady-state photosynthetic traits of a plant species cannot be used to predict the leaf behavior of that species in fluctuating light. My results have added to these tenets, by showing that the dynamic photosynthetic traits in fluctuating light, for experimental leaves that were constructed with heterogeneous PPFD pulses, did not express fidelity with those traits for the experimental leaves that were constructed with homogeneous shade. Reliable testing methods form a fundamental principle of scientific research, and these findings indicate that sunfleck research using leaves that were grown under homogeneous PPFD may exhibit minimal ecological relevance. In other words, much of the published data on leaf response to fluctuating light are artifacts created by inappropriate experimental methods employed to construct the experimental leaves.

Several other studies used analogous growth conditions by providing a similar integrated light level supplied as homogeneous versus heterogeneous light during experimental plant growth [27,28,29]. However, the response variables were focused on steady-state or integrated growth traits, so the results were not informative for directly comparing with my objectives. The only report which included photosynthetic induction as a response variable used an extremely low PPFD compared to my methods, and did not use a homogeneous approach among the treatments for determining the induction traits [27]. For example, the light flecks that the experimental leaves experienced during the construction prior to the photosynthetic induction measurements exhibited a maximum PPFD of only 280 µmol·m^−2^·s^−1^, a PPFD that was less than the shade flecks in my study. To my knowledge, this is the first report to use homogeneous versus heterogeneous shade conditions for growing the plants of several species to determine the resulting priming and induction responses to fluctuating light.

### 3.1. Looking Backward, Looking Forward

Refutation is an essential component of science. When new knowledge that informs experimental methods becomes available, continuing research must be designed in a manner that acknowledges the new information. Looking forward, this new knowledge establishes the mandate that appropriate leaves must be employed for use in research designed to determine leaf responses to fluctuating light. In laboratory settings, this could be accomplished with automated timers on the lamps of varied PPFD that provide experimental leaves with rapid transitions of fluctuating light throughout their expansion and maturation. In nursery settings, this could be accomplished with a slat-house structure similar to the methods reported herein, or motorized baffles that move to intermittently obstruct the incident light [27]. Alternatively, a sunfleck growth environment could be supplied by placing experimental plants underneath the canopy of shade trees in the nursery setting. The most defendable methods would be those of Kursar and Coley [30], where containerized plants were placed in situ such that the experimental leaves developed in light conditions that were ecologically relevant prior to the transfer of the plants to the controlled experimental setting.

Looking backward, when established experimental methods are shown to be inaccurate, what does one do with the established literature containing published artifacts [31]? Cleary, historical studies on photosynthetic induction should be revisited. In cases where inappropriate leaves were employed, the reliability of the resulting interpretations should be re-evaluated. One approach would be to determine if a universal transformation could be identified to calculate the corrections to each published data set. This approach would require every species to exhibit a similar amplitude and direction of differences between the homogeneous shade leaves and heterogeneous shade leaves. Unfortunately, the differences in trait covariation syndromes revealed by my three model species exhibited the complex phenotypic responses of plants to fluctuating light with regard to leaf construction under homogeneous versus heterogeneous shaded conditions.

The difference between the sun and shade leaves was over-estimated for some response variables, when the homogeneous shade leaves were used instead of heterogeneous shade leaves. For example, using homogeneous shade leaves artificially exaggerated the influence of shade (when compared with full sun) on A_max_, LMA, and the macronutrient concentrations in comparison to heterogeneous shade leaves. Alternatively, the difference between the sun and the shade leaves was under-estimated for some response variables when the homogeneous shade leaves were used. For example, using homogeneous shade leaves artificially minimized the influence of shade on the speed of priming, induction during first the 5 min, and IT90% in comparison to heterogeneous shade leaves.

A comparison to the response variables among the three model species provides a second approach for considering the complexity of leaf responses to developmental light conditions. For example, the three non-linear model coefficients, potassium concentration, and LMA significantly differed between the homogeneous and heterogeneous shade-grown leaves for maize and soybean, but not for cycad. In contrast, the calcium concentration, magnesium concentration, carbon/phosphorus, and carbon/potassium differed between the homogeneous and heterogeneous shade-grown leaves for maize leaves, but not for soybean or cycad.

The response variable that revealed the greatest disparity among the three model species was the number of light flecks that the homogeneous shade leaves required to reach the maximum Pn achievable within sequential 60 s light flecks. The amplitude of the maximum Pn within the fully primed light flecks differed among the species, but full sun leaves required six light flecks and heterogeneous shade leaves required three light flecks to reach the fully primed status for all three species. In contrast, the soybean leaves required four, cycad leaves required five, and the maize leaves required six light flecks to reach the fully primed status for the leaves that were constructed in the unnatural homogeneous shade conditions.

These collective results indicated that there was no universal relationship among the plant species concerning how the light environment during leaf construction influenced the mature leaf responses to fluctuating light. Clearly, if the experimental objective was to determine the influence of fluctuating light on leaf physiology, the safe experimental approach will ensure that the leaves are constructed in fluctuating light in preparation for the dates of data collection.

### 3.2. Complications

Fluctuating light does not influence leaf behavior in isolation of other abiotic factors. For example, the speed of stomatal response to fluctuating light that occurred in drought conditions was muted in well watered conditions [32]. The more rapid relaxation of Pn during shade flecks following light flecks occurred the under conditions of a water deficit, which indicated that the decreases of integrated carbon gains during the drought stress were more severe under the conditions of fluctuating light when compared to the conditions of uniform irradiances [33]. The speed of the photosynthetic induction in light flecks can be reduced by nitrogen deficiency [34], indicating that the plant nutrition directly influences the leaf physiology under fluctuating light conditions. Salinity stress may reduce plant growth and photosynthesis to a greater degree under the conditions of fluctuating light than under conditions of homogeneous light [35]. Global change conditions of elevated CO_2_ may enhance the photosynthesis and growth of plants to a greater degree under the conditions of fluctuating light than under the conditions of uniform irradiance [36].

When light is attenuated beneath tree canopies, the heterogeneity in light quantity occurs in combination with heterogeneity in light quality. Understory plants perceive and interpret these changes in the spectral composition of light as part of the information that is used to modify leaf behavior [37,38,39]. Differences in light quality may affect the photosynthetic responses of leaves to fluctuating light [40]. My methods focused on light quantity, but the influences of light quality on the response variables should also be studied for the leaves that were constructed under homogeneous versus heterogeneous incoming light conditions.

These interacting abiotic factors that directly influenced how plants responded to fluctuating light reveals the importance of context-dependency when conducting light-fleck research. For example, most published studies have not mentioned the nitrogen status of the experimental plants. Moreover, most reports included study plants that were grown under benign conditions with no stressors, and no mention was made of how common abiotic stressors may modify the outcomes.

## 4. Materials and Methods

### 4.1. Experimental Conditions

This study was conducted in a container nursery in Angeles City, Philippines, in 2018 and 2019. Three incident light treatments were created in the full sun nursery setting by the construction of shade houses with a semicircular shape. Polyvinyl chloride pipe with a 21 mm outside diameter was used for the framing. Each structure was 1.9 m in height and the curved sides were oriented East and West such that the incident solar beam was perpendicular to the light exclusion material for the entire photoperiod. This ensured that the percent of light exclusion was not influenced by the time of day. The footprint of each structure was 11.4 m^2^. A homogeneous shade treatment was provided with a commercial shade screen providing 62% light exclusion. A heterogeneous shade treatment designed to match the percent light exclusion of the shade screen was created by constructing a slat house with 5 cm wood slats positioned at a homogeneous distance in order to provide gaps through which the solar beam could pass. Diffuse light [41] and penumbral effects [42] added photosynthetically active radiation within the shaded areas provided by the slats, so the quantification of the integrated 62% overall light exclusion was achieved by adjusting the diameter of the gaps during construction until the desired PPFD was achieved. On the days with clear sky conditions and no clouds, 62% light exclusion was 780 µmol·m^−2^·s^−1^. For this purpose, integrated PPFD was quantified directly with a 0.75 m line quantum sensor (EMS-7, PP Systems, Amesbury, MA, USA) that was positioned perpendicular to the slats. The structure’s open North and South sides were covered with the shade cloth to ensure that the diffuse indirect light could not enter the structures. A full sun treatment was also used such that the study had three light treatments: homogeneous shade, heterogeneous shade, and full sun.

The plant material was grown in 2.6 L containers and the replications were grown sequentially rather than simultaneously. This approach removed the problem of pseudo-replication caused by only one shade structure per treatment in the experimental site. The cycad replications were selected from a container nursery comprised of Yap-sourced one-year-old seedlings. This species exhibits lengthy periods of time separating intermittent leaf growth. The population of containerized plants was inspected weekly beginning January 2018 to find the individuals with impending leaf emergence and growth. Whenever six seedlings with simultaneous leaf initiation were found, they were transferred to the experimental site and two were placed in each of the light treatment locations. On this date, the seeds of ‘Silver Queen’ maize and ‘Midori Giant’ soybean were planted in six containers per species to provide two plants of the three model species within each light treatment. Six seeds were planted per container, and they were thinned to one plant per container after seedling emergence. The cycad leaf growth required 17–22 days to reach the full expansion, and the leaf measurements were conducted one to two weeks later. At this time the maize and soybean plants exhibited numerous true leaves but no flowering. Four replications were initiated on the 10 March 2018, 7 June 2018, 3 November 2018, and 20 January 2019. The corresponding physiology measurements were conducted on 10–13 April 2018, 7–10 July 2018, 5–7 December 2018, and 20–22 February 2019.

The growing medium consisted of locally sourced washed river sand, and the plants were irrigated daily. The plant nutrition was provided by weekly drenches of 100 mL per container of a complete nutrient solution (Excel, Everris, North Charleston, SC, USA) with a concentration to provide 7.5 mM nitrogen. The cycad plants had received this weekly fertilization protocol since seed germination.

### 4.2. Physiology Measurements

The protocols for the leaf gas exchange were patterned after Marler [26]. The carbon dioxide leaf gas exchange was quantified with a CIRAS-2 portable open-flow system fitted with a PLC6(U) cuvette (PP Systems, Amesbury, MA, USA). Each plant that was intended for measurements was transferred to a covered open-air laboratory the night before the measurements. From sunrise until the time of the morning that each plant was used, the PPFD was ≈55 µmol·m^−2^·s^−1^. One plant per species was employed to quantify the photosynthetic response to sequential light flecks to determine the extent of priming that occurred. The homogeneity of the light flecks was ensured by the use of a light bank comprised of fluorescent and quartz halogen bulbs to provide a PPFD of ≈800 µmol·m^−2^·s^−1^. Each replication was initiated by placing the laminae in the cuvette for about 5 min to reach a steady-state gas exchange. The Pn was recorded about every 20 s for a period of 23–24 min. During this period, the leaf was subjected to 60 s light flecks separated by 120 s of shade flecks. The shade flecks were characterized by the diffuse shaded conditions with a PPFD of 55 µmol·m^−2^·s^−1^ in the ambient laboratory.

The measurements for the purpose of modeling the characteristics of photosynthetic induction were conducted on the second plant for each species. The leaves for these measurements were adjusted to the steady-state PPFD of 55 µmol·m^−2^·s^−1^ when they were inserted into the cuvette then abruptly subjected to a PPFD of 800 µmol·m^−2^·s^−1^ from the light bank for up to 40 min. The Pn was recorded every 30–60 s until 23–24 min. The cuvette was removed but the experimental leaves were maintained at a PPFD of 800 µmol·m^−2^·s^−1^. The Pn measurement was repeated at 30 and 40 min to ensure that the maximum net CO_2_ assimilation was measured. For every replication, the Pn at the end of the measurements for calculating the induction curve was homogeneous with the measurements at 30 and 40 min.

The chamber conditions were similar among all of the light fleck and the induction measurements because a controlled laboratory setting was used. The reference carbon dioxide was 400 µmol·mol^−1^, the air flow rate was 250 mL·min^−1^, the temperature was 28–31 °C, and hte vapor pressure deficit was 1.1–1.5 kPa.

### 4.3. Leaf Mass per Area and Nutrient Relations

The leaves or leaflets used for the physiology measurements were harvested from each plant. The leaf mass for the standardized area was calculate by cutting sections of each lamina to avoid any major veins. The lamina sections were trimmed into rectangular shapes and the sides of each rectangle were measured directly to the nearest mm. Lamina sections were placed in a forced draft oven at 75 °C for 24 h and weighed. The leaf mass per area was calculated from the area and dry weight. The sections used for the LMA were added back to the leaf samples obtained from each plant. The leaves from both plants per species per replication were combined into one sample per replication.

The combined leaf tissue for each replication was dried at 75 °C for 24 h, then milled to pass through a 20-mesh screen. Total nitrogen and carbon were determined by dry combustion (FLASH EA1112 CHN Analyzer, Thermo Fisher, Waltham, MA, USA) [43]. Samples were also digested by a microwave system with nitric acid and peroxide, then the macronutrients phosphorus, potassium, calcium, magnesium, and sulfur were quantified by inductively coupled plasma optical emission spectroscopy (Spectro Genesis; SPECTRO Analytical Instruments, Kleve, Germany) [44]. In addition to the macronutrient concentrations, the quotients carbon/nitrogen, carbon/phosphorus, and carbon/potassium were calculated to understand the influence of the developmental light on stoichiometry.

### 4.4. Calculations and Statistics

Photosynthetic induction results were handled as described by Marler [26]. The curves were fitted with the PROC NLIN procedure of SAS (SAS Institute Cary, NC, USA) with the model Pn = a + (b × time) + (c × log(time)) where the log was natural log. The data from each species and replication were fitted separately. Each of the three coefficients from the induction model and all the other response variables were subjected to analysis of variance to determine differences among the three developmental light treatments for each species. The stoichiometry variables were log-transformed prior to analysis.

## 5. Conclusions

Context dependency and appropriate experimental protocols are required to generate repeatable methods, dependable results, and accurate interpretations during biological research efforts. The methods employed during much of the light-fleck gas exchange research in the past 30 years has not adhered to these requirements. Interacting abiotic factors that directly influence how plants respond to fluctuating light should be controlled and reported, and the use of ecologically relevant experimental leaves for the data collection is needed. My results indicate that collecting leaf physiology data under fluctuating light using leaves that were constructed under homogeneous light such as commercial shade screen conditions is an ill conceived experimental approach that is prone to generating dubious outcomes.

## Figures and Tables

**Table 1:** Leaf photosynthetic traits of the three spermatophyte species following plant growth in homogeneous 62% sunlight exclusion under a shade screen, heterogeneous 62% sunlight exclusion under a slat-house, or full sunlight. The traits include the coefficients defining the induction model Pn = a + (b × time) + (c × log(time)) where log is the natural log, the net CO_2_ assimilation following full induction (A_max_), and the time to reach 50% (IT 50%) or (IT 90%) of the full induction. Mean ± SE, n = 4.

Trait	Homogeneous Shade	Heterogeneous Shade	Full Sun	*F*_2,9_	*p*
		*Cycas micronesica*			
Coefficient a	1.87 ± 0.21a	1.93 ± 0.22a	0.96 ± 0.21b	6.881	0.015
Coefficient b	−0.10 ± 0.02b	−0.09 ± 0.03b	0.20 ± 0.06a	21.223	0.001
Coefficient c	2.85 ± 0.08a	3.07 ± 0.21a	2.05 ± 0.11b	7.144	0.014
A_max_ (µmol·m^−2^·s^−1^)	8.50 ± 0.18c	9.63 ± 0.23b	11.25 ± 0.27a	47.860	<0.001
IT 50% (min)	3.23 ± 0.46b	3.25 ± 0.43b	6.13 ± 0.51a	15.171	0.001
IT 90% (min)	9.63 ± 0.72b	7.75 ± 0.46c	13.13 ± 0.81a	16.160	0.001
		*Glycine max*			
Coefficient a	8.90 ± 0.23b	10.36 ± 0.22a	5.50 ± 0.37c	57.551	<0.001
Coefficient b	−0.01 ± 0.06b	−0.29 ± 0.06c	0.66 ± 0.08a	56.685	<0.001
Coefficient c	4.05 ± 0.37b	7.00 ± 0.40a	3.73 ± 0.31b	24.762	0.001
A_max_ (µmol·m^−2^·s^−1^)	21.01 ± 0.61c	25.75 ± 0.63b	30.50 ± 0.87a	56.998	<0.001
IT 50% (min)	1.80 ± 0.17b	1.74 ± 0.13b	6.31 ± 0.31a	142.101	<0.001
IT 90% (min)	10.58 ± 0.82b	8.75 ± 0.66c	14.45 ± 0.88a	16.250	0.001
		*Zea mays*			
Coefficient a	9.48 ± 0.18b	10.35 ± 0.14a	4.77 ± 0.18c	197.121	<0.001
Coefficient b	0.03 ± 0.07b	−0.50 ± 0.05c	0.70 ± 0.08a	70.279	<0.001
Coefficient c	4.70 ± 0.61b	10.05 ± 0.38a	5.38 ± 0.29b	42.101	<0.001
A_max_ (µmol·m^−2^·s^−1^)	23.89 ± 0.61c	31.03 ± 0.80b	34.75 ± 0.75a	63.982	<0.001
IT 50% (min)	2.08 ± 0.83b	1.84 ± 0.44b	5.95 ± 0.46a	188.271	0.001
IT 90% (min)	11.11 ± 0.18b	8.58 ± 0.17c	13.98 ± 0.16a	20.078	<0.001

Means followed by the same letter within rows are not significantly different according to Tukey’s honest significant difference test.

**Table 2:** Elemental concentrations and stoichiometry of the *Cycas micronesica* leaves following plant growth in homogeneous 62% sunlight exclusion under a shade screen, heterogeneous 62% sunlight exclusion under a slat-house, or full sunlight. Mean ± SE, n = 4.

Trait	Homogeneous Shade	Heterogeneous Shade	Full Sun	*F*_2,9_	*p*
Carbon (mg·g^−1^)	475.12 ± 9.71	484.73 ± 9.60	483.33 ± 10.98	0.305	0.745
Nitrogen (mg·g^−1^)	37.11 ± 1.61a	28.320.79b	22.650.67c	49.660	<0.001
Phosphorus (mg·g^−1^)	2.89 ± 0.09a	2.62 ± 0.08b	2.82 ± 0.06a	5.531	0.027
Potassium (mg·g^−1^)	16.38 ± 0.53	14.90 ± 0.67	14.85 ± 0.49	2.462	0.140
Carbon/Nitrogen	12.91 ± 0.61c	17.14 ± 0.48b	21.37 ± 0.50a	108.88	<0.001
Carbon/Phosphorus	164.92 ± 3.60	185.01 ± 5.55	171.64 ± 5.18	3.823	0.063
Carbon/Potassium	29.06 ± 0.63	32.65 ± 0.95	32.68 ± 0.96	3.468	0.077
Calcium (mg·g^−1^)	3.12 ± 0.07a	2.80 ± 0.06ab	2.54 ± 0.05b	22.464	0.001
Magnesium (mg·g^−1^)	2.30 ± 0.05	2.21 ± 0.04	2.38 ± 0.06	2.565	0.131
Sulfur (mg·g^−1^)	1.64 ± 0.04a	1.21 ± 0.04b	1.26 ± 0.03b	40.880	<0.001

Means followed by the same letter within rows are not significantly different according to Tukey’s honest significant difference test.

**Table 3:** Elemental concentrations and stoichiometry of the *Zea mays* leaves following plant growth in homogeneous 62% sunlight exclusion under a shade screen, heterogeneous 62% sunlight exclusion under a slat-house, or full sunlight. Mean ± SE, n = 4.

Trait	Homogeneous Shade	Heterogeneous Shade	Full Sun	*F*_2,9_	*p*
Carbon (mg·g^−1^)	420.75 ± 5.14	427.75 ± 3.49	430.50 ± 4.44	0.2.180	0.169
Nitrogen (mg·g^−1^)	39.63 ± 1.67a	34.63 ± 1.58b	29.23 ± 1.59c	24.286	<0.001
Phosphorus (mg·g^−1^)	8.69 ± 0.16a	6.59 ± 0.13b	5.57 ± 0.15c	145.55	<0.001
Potassium (mg·g^−1^)	36.90 ± 0.79a	28.53 ± 0.46b	21.19 ± 0.69c	49.93	<0.001
Carbon/Nitrogen	10.63 ± 0.28c	12.37 ± 0.25b	14.88 ± 0.92a	14.91	0.001
Carbon/Phosphorus	48.40 ± 0.38c	65.03 ± 1.32b	77.52 ± 2.03a	106.58	<0.001
Carbon/Potassium	11.41 ± 0.22c	15.01 ± 0.23b	20.67 ± 0.49a	28.311	<0.001
Calcium (mg·g^−1^)	5.39 ± 0.06a	5.13 ± 0.05b	3.72 ± 0.09c	125.41	<0.001
Magnesium (mg·g^−1^)	3.02 ± 0.09c	3.39 ± 0.08b	3.89 ± 0.10a	21.590	<0.001
Sulfur (mg·g^−1^)	3.82 ± 0.06a	3.29 ± 0.10b	2.12 ± 0.07c	116.58	<0.001

Means followed by the same letter within rows are not significantly different according to Tukey’s honest significant difference test.

**Table 4:** Elemental concentrations and stoichiometry of the *Glycine max* leaves following plant growth in homogeneous 62% sunlight exclusion under a shade screen, heterogeneous 62% sunlight exclusion under a slat-house, or full sunlight. Mean ± SE, n = 4.

Trait	Homogeneous Shade	Heterogeneous Shade	Full Sun	*F*_2,9_	*p*
Carbon (mg·g^−1^)	400.42 ± 11.63	404.08 ± 10.91	400.70 ± 9.27	0.043	0.958
Nitrogen (mg·g^−1^)	63.87 ± 1.17a	53.91 ± 1.79b	53.13 ± 1.61b	17.310	0.001
Phosphorus (mg·g^−1^)	11.23 ± 0.48a	9.41 ± 0.24b	10.12 ± 0.64a	5.874	0.023
Potassium (mg·g^−1^)	43.55 ± 1.61a	38.13 ± 1.40b	36.68 ± 1.28b	6.345	0.019
Carbon/Nitrogen	6.28 ± 0.27b	7.49 ± 0.44a	7.56 ± 0.39a	5.107	0.033
Carbon/Phosphorus	35.97 ± 2.39	44.94 ± 2.23	39.70 ± 2.71	3.316	0.083
Carbon/Potassium	9.92 ± 0.55	10.61 ± 0.64	10.97 ± 0.65	3.389	0.080
Calcium (mg·g^−1^)	17.25 ± 1.42	14.88 ± 0.90	14.13 ± 0.94	2.468	0.140
Magnesium (mg·g^−1^)	8.55 ± 1.78	8.43 ± 2.11	9.13 ± 2.18	0.034	0.967
Sulfur (mg·g^−1^)	6.23 ± 0.27a	4.95 ± 0.43b	5.95 ± 0.35a	4.842	0.037

Means followed by the same letter within rows are not significantly different according to Tukey’s honest significant difference test.
